# Editorial: Nocebo Effects and Their Influence on Clinical Trials and Practice: Modulating Factors in Healthy and Pathological Conditions

**DOI:** 10.3389/fphar.2020.00100

**Published:** 2020-02-19

**Authors:** Martina Amanzio, Lene Vase, Dimos D. Mitsikostas

**Affiliations:** ^1^ Department of Psychology, University of Turin, Turin, Italy; ^2^ European Innovation Partnership on Active and Healthy Ageing, Bruxelles, Belgium; ^3^ Department of Psychology and Behavioural Sciences, School of Business and Social Sciences, Aarhus University, Aarhus, Denmark; ^4^ First Department of Neurology, Aeginition Hospital, National and Kapodistrian University of Athens, Athens, Greece

**Keywords:** nocebo effect and nocebo responses, neurological diseases, schizophrenia, chemotherapy, muscular performance, expectation theory

The nocebo phenomenon comprises negative health outcomes following negative perceptions, communications and expectations toward experimental paradigms and treatment outcome. Recently, the nocebo phenomenon has become a hot topic as it may compromise the outcome of treatments and adherence to therapy in both clinical trials and practice.

The present editorial will focus on the published articles characterizing the nocebo phenomena in clinical trials, experimental studies and literature reviews. These findings will help hypothesize significant changes in clinical trials and clinical practice by highlighting the importance of best communication strategies in personal care, taking into account the individual subject`s predispositions and the doctor-patient relationship in order to tailor an individualized treatment approach.

Nocebo responses will be presented in a mini review of functional MRI pain anticipation-experimental-paradigms, and nocebo effects will be reviewed in an evaluation of adverse events in the placebo groups of randomized control trials (RCTs) in neurological and psychiatric disorders. Nocebo effects are also described in subjects with post-chemotherapy nausea and the potential impact on muscular performance is presented. Finally, the influence of communication on nocebo effects is discussed.

This editorial is a brief overview of the contributions to our Research Topic and we encourage a careful reading of the published studies for a more detailed understanding of nocebo effects and responses.

## Nocebo Responses in Pain Anticipation fMRI Paradigms

In a mini review, Amanzio and Palermoz described the results from fMRI studies in human pain experimental models in order to explain the mechanisms underlying nocebo responses.

Regarding neurofunctional pain anticipation mechanisms, data show neural activations of cortical systems associated with pain experience, even without nociceptive stimulations. As these cerebral areas are involved in the selection of sensory (pain/interoception), attentional and emotional resources, they are considered crucial in terms of cognitive control signals. In fact, potentially dangerous stimuli, such as painful ones, elicit processes, which generate an escape motivation fundamental for individual survival mechanisms (Palermo et al.).

In case of neurodegenerative disorders, such as Alzheimer's Disease or behavioural frontotemporal dementia, a disrupted neurocognitive anticipatory network caused by pain modulatory system impairment, could be responsible for pain-processing alterations (Amanzio and Palermo). Indeed, some studies have shown an association between damage of prefrontal control and a poor response to analgesic therapies and the placebo effect, leading to the need to administer higher doses of drugs with analgesic action in these patients (Defrin et al.).

## Nocebo Effects in RCTs of Placebo Groups in Patients With Neurological Disorders and Psychiatric Diseases

Spanou et al. described nocebo effects in RCTs of placebo groups, considering studies and trials regarding neurological diseases in adults. The authors concluded that nocebo effects exist and can affect the efficacy and tolerability of generics and biosimilars. Nevertheless, future studies are needed in order to better understand this occurrence and limit its negative consequences.

Palermo et al. described nocebo effects in RCTs of placebo groups in patients with schizophrenia spectrum disorders. Using the positive and negative syndrome scale, they found a relationship between psychiatric symptoms and the rates of adverse events (AEs) reported as nervous system and gastrointestinal disorders in the placebo arms of double-blind clinical trials. Furthermore, they observed a positive association between psychiatric symptomatology and AEs, making patients more inclined to develop negative outcomes (Hwang et al.).

## Nocebo Effects in Patients With Post-Chemotherapy Nausea and on Muscular Performance

Meissner et al. studied the variables influencing nausea expectancy and its actual occurrence in female patients undergoing chemotherapy for cancer. They discovered that younger age, lower quality of life, and prior experience with nausea could be considered as predisposing factors of post-treatment nausea in first-time chemotherapy patients. These data are important for medical settings as the risk of post-treatment nausea should be further reduced. Moreover, antiemetic drugs and psychological co-interventions could help reduce nausea and improve adherence to chemotherapy.

In order to observe possible nocebo effects, Zech et al. examined whether verbal and non-verbal communication—words, sentences, images, etc.—could affect muscular strength. They measured the maximal arm muscular strength after making positive and negative suggestions from medical setting and daily clinical practice (for instance, risk information for informed consent). Presenting verbal and non-verbal nocebo-stimuli had a weakening impact on muscular performance in volunteers. In particular, the authors underlined that these nocebo effects are to be considered a direct consequence of verbal and non-verbal communication rather than a product of mental or psychological interventions (Fiorio et al.). In addition, due to negative suggestions, the impaired muscular performance could be part of a more general “weakening effect” (Zech et al.).

## Communication Strategies in Personal Care, Individual Subjects’ Predispositions in the Doctor–Patient Relationship

Barnes et al. described the importance of positive communication strategies used in order to reduce nocebo side effects while maintaining informed consent instructions. They focused their attention on framing—the way in which information is presented, of AEs in terms of significance or likelihood. Positive valence framing of side effect warnings (on their aspects of experience, attribution or threat) seemed to attenuate anticipatory anxiety, attention to negative symptoms, and, consequently, nocebo effects. Nevertheless, future research should address different types of framing effects and psychological mechanisms underlying framing effects, which could be relevant in order to achieve the best outcome.

Hansen and Zech observed how specific types of possible negative suggestions, in the presentation of informed consent, are involved in eliciting nocebo effects and presented different approaches in order to avoid them. One method could be offering different opportunities rather than denying issues or lying, which should minimize the possibility of negative results (for instance, giving information not only on risks but also on the advantages of treatments). The authors concluded that additional explaining models could be useful for a better understanding of negative aspects of communication considered as nocebo effects.

## Author Contributions

MA wrote the first draft of the Editorial. LV and DM have made a substantial, direct, and intellectual contribution to the work, and approved it for publication.

## Conflict of Interest

The authors declare that the research was conducted in the absence of any commercial or financial relationships that could be construed as a potential conflict of interest.

